# Deep Segmentation of Point Clouds of Wheat

**DOI:** 10.3389/fpls.2021.608732

**Published:** 2021-03-24

**Authors:** Morteza Ghahremani, Kevin Williams, Fiona M. K. Corke, Bernard Tiddeman, Yonghuai Liu, John H. Doonan

**Affiliations:** ^1^National Plant Phenomics Centre, Institute of Biological, Environmental and Rural Sciences, Aberystwyth University, Aberystwyth, United Kingdom; ^2^Department of Computer Science, Aberystwyth University, Aberystwyth, United Kingdom; ^3^Department of Computer Science, Edge Hill University, Ormskirk, United Kingdom

**Keywords:** 3D analysis, segmentation, convolutional neural network, deep learning, pattern, point cloud, wheat

## Abstract

The 3D analysis of plants has become increasingly effective in modeling the relative structure of organs and other traits of interest. In this paper, we introduce a novel pattern-based deep neural network, Pattern-Net, for segmentation of point clouds of wheat. This study is the first to segment the point clouds of wheat into defined organs and to analyse their traits directly in 3D space. Point clouds have no regular grid and thus their segmentation is challenging. Pattern-Net creates a dynamic link among neighbors to seek stable patterns from a 3D point set across several levels of abstraction using the K-nearest neighbor algorithm. To this end, different layers are connected to each other to create complex patterns from the simple ones, strengthen dynamic link propagation, alleviate the vanishing-gradient problem, encourage link reuse and substantially reduce the number of parameters. The proposed deep network is capable of analysing and decomposing unstructured complex point clouds into semantically meaningful parts. Experiments on a wheat dataset verify the effectiveness of our approach for segmentation of wheat in 3D space.

## 1. Introduction

Three- and four-dimensional phenotyping has the potential to provide reliable, comprehensive information on morphological and developmental traits in plants. With recent improvements in image acquisition and 3D reconstruction, future studies would benefit from rapidly assessing 3D models (Chaudhury et al., 2018; Bernotas et al., 2019; Chaudhury and Godin, 2020; Artzet et al., under review). Accurate 3D models enable quantitative analyses of various traits, and a high-throughput spatial and temporal 3D analysis tool could monitor impacts of different treatments in experiments and, ultimately, management decisions in production conditions. 3D or higher-order data, however, requires complex processes for both acquisition and computation while quality can vary due to numerous factors such as imaging noise, occlusion, spikes, holes, lack of homogeneity, and interference from cluttered backgrounds. Despite the obvious attractions, few segmentation techniques have been reported for 3D point clouds of plants and they tend to require specific conditions that cannot easily be generalized.

Wheat is globally important with more than 700 million tonnes of grain produced annually (FAO report 2020)^1^. The grain-filling period of wheat is a key growth period that directly influences yield. There is widespread interest in estimating the number of ears per unit area (Ferrante et al., 2017) and other traits crucial for determining yield from images. Ear segmentation is therefore critical in estimating yield in wheat (Bi et al., 2010; Kun et al., 2011; Chen et al., 2016; Alharbi et al., 2018; Tan et al., 2020). Manual data collection, involving visual inspection of the standing crop, is labor intensive and time-consuming. Image processing and computer vision techniques facilitate high-throughput counting of ears. Such techniques can rapidly estimate yield, potentially accurately and with minimal human intervention.

Deep learning has been invaluable for the development of high-throughput pipelines that undertake 2D image analysis of wheat and many other plants (Qiongyan et al., 2017; Hasan et al., 2018; Wang X. et al., 2019; Hamidinekoo et al., 2020). Learning methods capable of extracting high-level features from raw input data with minimal human intervention would be useful for high-throughput pipelines. Lack of depth information is a major drawback of current 2D imaging, limiting the accurate quantitative evaluation of many traits. In this study, we demonstrate that deep learning techniques can also be used to directly segment 3D geometric wheat data, acquired using standard 3D structure from motion techniques (Furukawa and Ponce, 2010; Jay et al., 2015; Schönberger and Frahm, 2016; Schönberger et al., 2016). In this report, we propose a novel network that efficiently handles highly complex 3D point clouds. Unlike most segmentation techniques that heavily rely on data and its distribution, our proposed network extracts stable patterns from point clouds across different levels of features obtained through the K-nearest neighbor algorithm. Our network is thus more robust to variation in the density of point cloud data, typical imaging distortions, and noise. To the best of our knowledge, this paper is the first study to segment and analyse ears directly within the point cloud domain via deep learning. The proposed framework has been validated using 690 wheat point clouds, captured at different times during the growth cycle. The results indicate that our deep learning method is robust and can accommodate irregular point clouds that are noisy and contain irrelevant outliers.

In section 2, we review previously reported segmentation techniques in plant science. The proposed pattern-based deep neural network (Pattern-Net) is detailed in section 3. Section 4 reports and discusses the experimental results of Pattern-Net on the wheat dataset. Section 5 relates our findings to previous studies and, finally, conclusions and future work are provided in section 6.

## 2. Background

Segmentation of ears is challenging due to their highly complicated and varied shapes and numbers and unpredictable interaction with their background. Most studies to date have been carried out in the 2D domain using standard images (Chopin et al., 2016; Zhou et al., 2018; Misra et al., 2020). A hybrid approach (Chopin et al., 2016) uses a-priori information about the shape of leaves and local image orientations to fit active contour models to features that are missed during the initial segmentation. Mohanty et al. (2016) applied a deep learning method for plant disease detection. Madec et al. (2019) employed a CNN to identify ears from low-spatial-resolution RGB images. Ubbens and Stavness (2017) implemented deep convolutional neural networks (CNNs), successfully estimating leaf number from an image database of Arabidopsis rosettes. Sadeghi-Tehran et al. (2019) developed a deep CNN-based classification technique to automatically identify and count the number of ears in images taken under natural field conditions. Recently, a 2D CNN model (Xu et al., 2020) extracted the contour features of ears using a K-means clustering algorithm and then classified the segmented images using a five-layered CNN. These examples clearly demonstrate the potential of these approaches to extract useful biologically relevant information from images and the feasibility of scaling to accommodate very large datasets.

Previous methods for segmenting point clouds considered constraints and used learning-based optimization techniques such as clustering, support vector machine (SVM) etc. (Paulus et al., 2013; Li et al., 2018). Gélard et al. (2017) segmented leaves using a geometrical constraint and Euclidean cluster extraction method. Liu et al. (2018) exploited a revised version of Euclidean distance and spectral clustering to segment individual leaves from a variety of plants including wheat. Multi-view vision segmentation techniques (Guo and Xu, 2017; Shi et al., 2019) have been applied to stereo multi-view 2D images. The performance of three learning methods including SVM, boosting, and K-means clustering in the segmentation of soybean plants were compared in Zhou et al. (2019), where K-means clustering outperformed the other methods in terms of processing efficiency and segmentation accuracy. We previously used a semi-automatic method for segmentation of leaf and petiole in Grapevine to quantify drought responses from images (Briglia et al., 2020). Jin et al. (2018) proposed an indirect method for 3D object detection and segmentation, whereby a region-based CNN (RCNN) is used to detect objects in 2D images projected from 3D points.

Since traditional point cloud-based segmentation methods consider some constraints that depend on traits of interest, the generalization of such methods is not straightforward. The efficiency of previous methods is also questionable in highly complex noisy 3D models. To address these drawbacks, a tensor-based technique has been developed that represents highly-complex models by their first- and second-order tensors without requiring pre-defined shape assumptions and constraints (Elnashef et al., 2019). Most recently, Li et al. (2019) employed a 3D joint filtering operator for leaf segmentation. Here, we introduce a new procedure for segmentation of 3D point cloud data from plants using deep neural networks.

A deep learning-based point cloud segmentation named PointNet (Qi et al., 2017a) has been recently proposed that is capable of extracting high-level features from raw input data via learning on sufficient 3D CAD models^2^ of various objects. The mean accuracy of PointNet is an impressive 84% and has been further improved by Qi et al. (2017b), Shen et al. (2018), Guerrero et al. (2018), Landrieu and Simonovsky (2018), and Wang Y. et al. (2019). Despite poor existing segmentation methods, 3D point cloud deep learning segmentation methods can effectively handle complex models across a wide array of species. Applying these techniques to typical 3D models of plants (>10^4^ points) is almost impossible since current GPU devices are unable to process such large models. These issues motivated us to further develop a light deep network for point cloud segmentation (Ghahremani et al., 2020) that is highly effective for architectural models. However, direct application of this method to plant point cloud data did not yield satisfactory results since plants tend to occupy volumetric space in a very different manner from buildings for example—with complex structures, configurations, occlusion, and often cluttered background. Here we expand our recent segmentation method (Ghahremani et al., 2020) to wheat point clouds. To the best of our knowledge, the proposed network provides the first practical segmentation of plant parts directly within the point cloud domain. We provide thorough empirical and theoretical analysis on the stability and efficiency of the proposed Pattern-Net method using more than 690 wheat point clouds and demonstrate its ability to extract biologically meaningful data in terms of accurate ear counts and ear-length estimates.

## 3. Proposed 3D Point Cloud Segmentation Network

The goal is to establish and train a deep neural network that converts an input point set *P* = {*p*_1_, …, *p*_*M*_} into a set of segmentation labels. Here, *M* denotes the total number of 3D points and they are represented as a set of 3D coordinates. The ground-truth label is a vector of length *M*, Γ = {γ_1_, …, γ_*M*_}, where γ_*i*_ is the label of *i*-th point. Since there are *N* segmentation labels, thus γ_*i*_ ≤ *N*. The output of the network is a vector of predicted labels, i.e., $\hat{\Gamma }=\left\{{\hat{\gamma }}_{1},\ldots ,{\hat{\gamma }}_{M}\right\}$. The principles of the proposed Pattern-Net are explained in the following sections. Ghahremani et al. (2020) provided more details about implementation.

### 3.1. Network Properties

A segmentation network for a point cloud set must meet the following four requirements about invariance (Qi et al., 2017a,b; Ghahremani et al., 2020):

*Property I (permutation invariance)*: This property states that the segmentation labels must be invariant to changes in the order of 3D points. If γ_*i*_ and γ_*j*_ are the segmentation labels of 3D points *p*_*i*_ and *p*_*j*_, respectively, then

(1) $$\begin{array}{r} \left[\gamma_{i},\gamma_{j}\right]=\left[\gamma_{j},\gamma_{i}\right],\;\forall i,j\in \left\{1,\ldots ,M\right\}, \end{array}$$

where [.] indicates the order. Unlike pixels in images or voxels in volumetric grids, a 3D point cloud set has no order and due to its irregular format, the segmentation network must be invariant to the order of the points.

*Property II (transformation invariance)*: The segmentation results must not be varied by changes in affine transformation, i.e.,:

(2) $$\begin{array}{r} \Gamma_{r\left[p_{1},\ldots ,p_{M}\right]+t}=\Gamma_{\left[p_{1},\ldots ,p_{M}\right]}. \end{array}$$

3D models may be captured or described under different viewpoints (rotation) and translations (position) at different growth time (scaling). These factors must not influence the segmented labels when a network segments a point cloud of interest.

*Property III (3D points relations)*: In point cloud domain the relationship between 3D points, denoted by *R*, is determined by their distance from each other:

(3) $$\begin{array}{r} R\left\{p_{i},p_{j}\right\}=D\left(p_{i},p_{j}\right). \end{array}$$

The distance metrics could be Euclidean distance, Manhattan distance, cosine distance, etc. Points in the point cloud domain are not isolated and their neighbors represent meaningful parts/organs that execute particular functions and produce particular behaviors.

*Property IV (resolution-invariance)*: The density of 3D points (or equivalently the number of points) must not influence the performance of the segmented regions. The density of the point cloud influences the relationship parameter defined in Equation (3), but the overall segmentation results must remain unchanged.

These four properties provide the foundation for the design of our network.

### 3.2. Network Architecture

**Figure 1:**
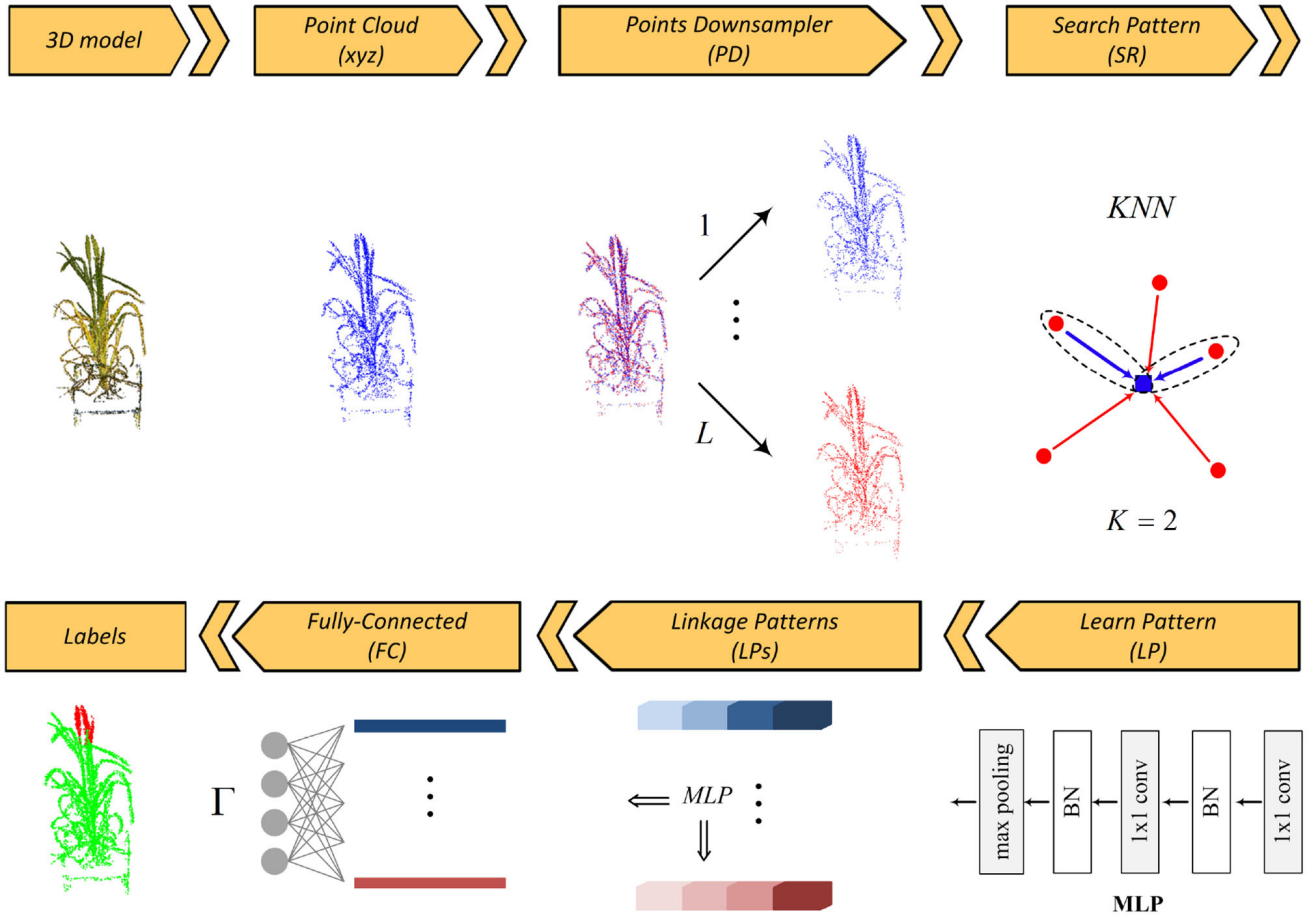
Pattern-Net architecture for segmentation of a point cloud of wheat.

The basic steps of the proposed segmentation network are depicted in Figure 1. The framework has five main layers: points downsampler (PD), search pattern (SP), learn pattern (LP), linkage patterns (LPs), and fully connected (FC) layers.

The input 3D point set is first decomposed into “*L*” levels by the PD layer. Inside each scale level, the relationship between each query point and its neighbors is sought by the KNN algorithm embedded in the SP layer and then is learned as a pattern by the LP layer. There are several interactions between the SP and the LP layers for extracting the deep patterns from the relationships of 3D points. The linkage features (LPs) layer links all learnt patterns across all levels and finally an FC layer predicts the segmentation labels. In the following, we detail these layers.

**Points Downsampling (PD) Layer**: Image acquisition is undertaken at different zoom levels and growth times that directly affect quality, density, and quantity of the point clouds. The function of this layer is to make the deep network independent of the quantity and distribution of points (*Property IV*). To this end, we decompose the input 3D point cloud, *P*, into *L* sets via a random downsampling operator, in such a way that all the 3D point subsets, *P*{*l*},  *l* ∈ {1, …, *L*}, are completely different while their overall schemes/abstracts are similar to each other:

(4) $$\begin{array}{r} P\left\{l\right\}\cap P\left\{k\right\}=\emptyset \;\forall l,k\in \left\{1,\ldots ,L\right\}, \end{array}$$

(5) $$\begin{array}{r} \overset{L}{\underset{l=1}{⋃}}P\left\{l\right\}=P, \end{array}$$

(6) $$\begin{array}{r} R\left\{l\right\}≃R\left\{k\right\}\;\forall l,k\in \left\{1,\ldots ,L\right\}. \end{array}$$

As illustrated in Figure 1, the segmented regions of a plant remain unchanged across different sampling levels while none of the decomposed point sets shares identical points. The main idea is to enforce the network keeping patterns of a 3D point cloud throughout all sampling levels. Similar organs/parts across different sampling levels share similar global features and this will assign considerable weights to such organs in the LPs layer, while dissimilar organs have smaller weights that are removed by a dropout operator. As will be discussed in “search pattern,” this strategy also effectively helps the network not to be saturated with its K nearest neighbors while keeping the radius of the neighborhood reasonable. In short, the main advantages of using multi-level sampling analysis are:

- Detection of hidden general patterns by decomposing a complex point cloud into simpler ones;
- Making a balance between the searching area and K responses; and
- Efficiently reducing the computational complexity of the KNN algorithm.

**Search Pattern (SP) Layer**: The task of this layer is to search all possible relationships between the query point/feature (**f**_*q*_ = (*f*_*q, x*_, *f*_*q, y*_, *f*_*q, z*_)) and its neighbors via the KNN algorithm (*Property III*). For each of K nearest neighbor responses (**f**_*i*_ = (*f*_*i, x*_, *f*_*i, y*_, *f*_*i, z*_), *i* = {1, 2, .., *K*}, *i* ≠ *q*), we compute all three possible edges emanating from the query point along three axes (i.e., **f**_*i*_ − **f**_*q*_), and stack it with the query point coordinates/feature **f**_*q*_. Thus, there is a feature space of size *K* × 6 for each query point. Adding edges to the feature space is important as KNN sorts K nearest responses and how far KNN responses are from the query point should be taken into account.

**Learn Pattern (LP) Layer**: The function of this layer is to find and to learn a meaningful relationship/pattern between all input 3D points via a two consecutive 2D convolution kernel followed by a batch normalization operator. A max-pooling operator is then applied to the output weights to get the features of the query point. The max-pooling is a symmetric function that guarantees that the extracted features are permutation-invariant (*Property I*). The combination of 2D convolution kernels, batch normalization and max-pooling operators is often called multi-layer perceptron (MLP) (Qi et al., 2017a). Inside each decomposed set, relationships between each query point and its neighbors are sought by the SP layer and then learned by the LP layer. This is done by applying and concatenating four MLPs {32, 32, 32, 32}, yielding from low-level features to high-level ones. Hence, there is a feature vector of length 128 for each 3D point inside each decomposed set.

**Linkage Patterns (LPs) Layer**: This layer contains several MLP layers and it aims to link the patterns that are similar across all the decomposed levels. As can be seen in the figure, the LPs layer is fed by all the low-level and high-level features. By applying a max-pooling operator to the features of the points inside a sampling set, a description vector of length 128 is obtained. We arrange all the local description vectors ψ_*l*_, *l* ∈ {1, …, *L*} in a matrix Ψ. We then apply an MLP to the whole cube features of the points to yield a global description vector ϕ. As discussed in section 3.3, the global description vector is used as a guideline for extracting stable patterns in the feature space.

**Fully-Connected (FC) Layer**: This layer functions as a decoder and maps the patterns extracted in the preceding layer into Γ labels. The output of the LPs layer is decoded by three consecutive MLPs {256, 256, Γ}. The drop-rate of all the decoding MLPs except the last one is fixed at $\frac{2}{3}$.

### 3.3. Network Loss Function

The goal of the LPs layer is to make the local vectors ψ_*l*_, *l* ∈ {1, …, *L*} as close to the global one ϕ as possible for the detection of the stable patterns inside the given point cloud. Assume that there is a linear relationship between the cloning and global description vectors, i.e., ϕ = Ψω, the estimated coefficients ω can be computed by the Moore–Penrose inverse (Penrose, 1955), i.e.,

(7) $$\begin{array}{r} \omega ={\text{Ψ}}^{†}\phi ={\left({\text{Ψ}}^{T}\text{Ψ}\right)}^{-1}{\text{Ψ}}^{T}\phi . \end{array}$$

The Moore–Penrose pseudo-inverse could be simply implemented by singular value decomposition (SVD) (Brake et al., 2019). The coefficient vector ω measures the contribution of each local set in the resulting global one. The variance σ(ω) of elements of ω approaches zero if all the local description vectors are close to the global one. We add this term into the loss function as follows:

(8) $$\begin{array}{r} L(\theta )=\underset{segmentation\;loss}{\underset{︸}{-\frac{1}{n}\sum_{i=1}^{M}\sum_{k=1}^{\Gamma }\Omega_{k}y_{ik}\log p_{ik}}}+\lambda \underset{linear\;mapping\;loss}{\underset{︸}{\sigma (\omega )}} \end{array}$$

In the above equation, the first term is the cross-entropy function for computing the loss of the predicted labels and the second term forces the network to yield zero standard deviation for the coefficients obtained by the linear mapping. *y*_*ik*_ is one-hot encoded labels and *y*_*ik*_ is scaled softmax logits. λ is a predetermined hyperparameter. In the segmentation of plants, some organs are of more interest than others; for example, the segmentation of ears is more important than those of the other organs. To deal with imbalanced distributions of organ-specific point clouds, we have added a dynamic coefficient vector, Ω, into Equation (8), which is defined as

(9) $$\begin{array}{r} \Omega_{k}=|C_{k}-\frac{\overset{M}{\underset{i=1}{\sum }}\left(\gamma_{i}==k\right)}{M}|, \end{array}$$

where *C*_*k*_ is a probability constant that determines the significance of the *k*-th segmented organ.

## 4. Experimental Results

### 4.1. Data Acquisition and Preparation

Spring wheat (variety Paragon) was used to acquire the images for modeling. These plants comprised part of Experiment W048 being undertaken to benchmark wheat growth under LED lighting. Briefly, they were grown as single plants in 1 L capacity pots containing Levington F2 peat-based compost. After germination, plants were grown on a conveyor based automated watering and imaging system (Lemnatec, Germany) at National Plant Phenomics Centre (NPPC)^3^ and grown under white LED Sunblaster (Kroptek, Sussex UK) luminaries at light level of 400 μMm^−2^s^−1^. Pots were watered daily to a target weight equivalent to either 75% (well-watered) or 35% (droughted) of field capacity and grown to maturity. The image acquisition system employed a pair of freestanding DSLR cameras in carefully calibrated locations that have been piggybacked onto the propriety LemnaTec platform, which acts as a delivery and lighting system for routine image collection. An in-line turntable was used to rotate subjects through 360 degrees and camera triggering was controlled and synchronized by prototype software, and image collection was based on commands from “gphoto2”^4^. Each image acquisition event provides 74 high-resolution multi-view images (6,000 × 4,000 px.) per plant. For the purposes of this analysis, we used images from 10 individuals grown under well-watered conditions and 10 individuals grown under drought, and a total of 690 point clouds were reconstructed and selected for segmentation.

The 3D models were reconstructed from the multi-view images by COLMAP (Schönberger and Frahm, 2016; Schönberger et al., 2016). COLMAP includes two phases: structure-from-motion (SfM) for sparse reconstruction and multi-view stereo (PMVS) for dense reconstruction. SfM extracts the calibration parameters including intrinsic and extrinsic parameters/matrices from the multi-view images. To this end, we detected keypoints from images by FFD (Ghahremani et al., 2021) and then extracted features from the keypoints by InterTex feature descriptor (Ghahremani et al., 2021). Exhaustive matching (Codreanu et al., 2013) was applied to the features to find corresponding keypoints in the multi-view images. The matched keypoints were then verified by geometric verification and finally, the structure and motion reconstruction were extracted (Schönberger and Frahm, 2016). PMVS (Schönberger et al., 2016) projected the 2D images into 3D space using the transformation matrices obtained by SfM and forms point clouds as outputs.

**Figure 2:**
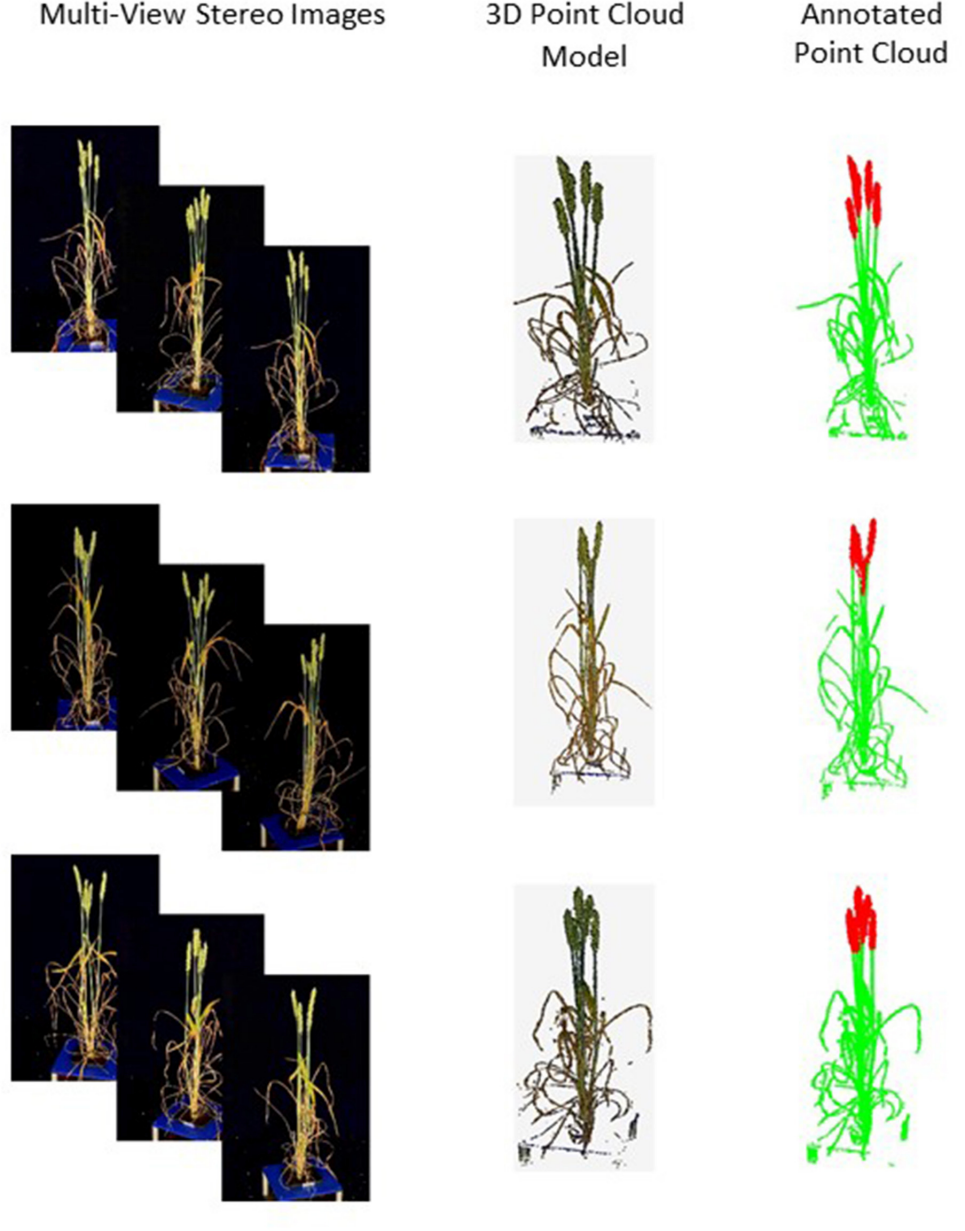
Samples of the captured multi-view images, their reconstructed point clouds and annotated ones. Ears in annotated point clouds are shown in red and non-ears in green.

We annotated the point clouds using MeshLab software (Ranzuglia et al., 2013). Regions of interest were extracted and labeled into one of two semantic categories—ear and non-ear. Thus, the number *N* of labels is equal to 2 and examples are shown in Figure 2. The segmentation task was repeated under a different number of input points ranging from 512 to 16,384. Final harvest measurements including plant height, ear number, and ear length were used for independent verification of the segmentation results.

### 4.2. Evaluation Metrics

The segmented point clouds were assessed by the mean intersection-over-union (*m*IoU) and mean accuracy (*m*A). These metrics are widely used for assessing segmentation results. According to commonly accepted definition, accuracy is the ratio of true predicted labels to the whole points and IoU is the number of points common between the labels (Γ) and predicted ones ($\hat{\Gamma }$) divided by the total number of points present across both the labels and predicted ones, i.e.,

(10) $$\begin{array}{r} IoU_{k}=\frac{\Gamma_{k}\cap {\hat{\Gamma }}_{k}}{\Gamma_{k}\cup {\hat{\Gamma }}_{k}},m\in \left\{1,\ldots ,N\right\}. \end{array}$$

**Figure 3:**
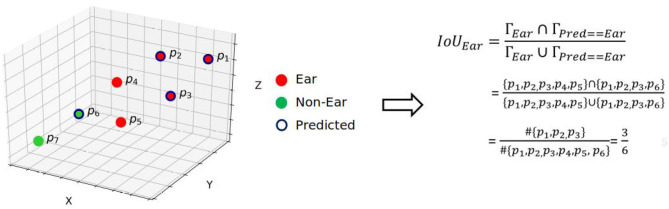
Computation of IoU for the predicted ears in the point cloud domain.

The procedure for computing IoU of ears is illustrated in Figure 3. The average of all the organs' IoUs, i.e., $\frac{\overset{N}{\underset{k=1}{\sum }}IoU_{k}}{N}$, yields the *m*IoU. We also assessed the segmentation results using Pearson correlation coefficient (*R*^2^) and root relative mean square (RRMSE):

(11) $$\begin{array}{r} RRMSE=\sqrt{\frac{1}{C}{\sum_{i=1}^{C}\left(\frac{B_{i}-{\hat{B}}_{i}}{B_{i}}\right)}^{2}}, \end{array}$$

where *B*_*i*_ is the ground-truth counted ears and ${\hat{B}}_{i}$ is the predicted ones. *C* is the total number of point clouds processed and it equals 690 in this study.

Given paired data $\left\{\left(B_{1},{\hat{B}}_{1}\right),\ldots ,\left(B_{i},{\hat{B}}_{i}\right)\right\}$ consisting of *C* pairs, Pearson correlation coefficient *R*^2^ is defined as:

(12) $$\begin{array}{r} R^{2}=\frac{\sum_{i=1}^{C}\left(B_{i}-\bar{B}\right)\left({\hat{B}}_{i}-\bar{\hat{B}}\right)}{\sqrt{\sum_{i=1}^{C}{\left(B_{i}-\bar{B}\right)}^{2}}\sqrt{\sum_{i=1}^{C}{\left({\hat{B}}_{i}-\bar{\hat{B}}\right)}^{2}}}, \end{array}$$

where

(13) $$\begin{array}{r} \bar{B}=\frac{1}{C}\overset{C}{\underset{i=1}{\sum }}B_{i},\bar{\hat{B}}=\frac{1}{C}\overset{C}{\underset{i=1}{\sum }}{\hat{B}}_{i}. \end{array}$$

### 4.3. Data Preparation for Training and Testing

The wheat dataset was randomly split into 580 training, 30 validation and 80 test samples. The code was implemented in TensorFlow 1.12 (Abadi et al., 2015) on a 64-bit computer with Intel(R) Xeon(R) Gold 6130 CPU @ 2.10 GHz processors, 48 GB RAM, and two Tesla P100-PCIE-16GB GPU devices. The entire model was trained by minimizing the loss function stated in Equation (8). We used the Adam optimization algorithm with a constant learning rate of 0.001, and we reduced the learning rate until 0.0001 using the exponential decay function. Since there exists a direct relationship between the complexity and the required GPU resources, we have also carried out the training procedure on a light version of Pattern-Net, called light Pattern-Net, where the size of MLPs is half of the Pattern-Net, i.e., 16. The batch size, hyperparameter λ and parameter *L* were set to 10, 10,000, and 8, respectively. Because of the agronomic importance of the ear, *C*_*ear*_ in Equation (9) was set to 1 and the other category, i.e., *C*_*non*−*ear*_, was set to 0.95. During the training step, the point clouds were augmented by randomly rotating, scaling and translating, in order to ensure that the network was transformation invariant, required by *Property II*.

### 4.4. Results

**Figure 4:**
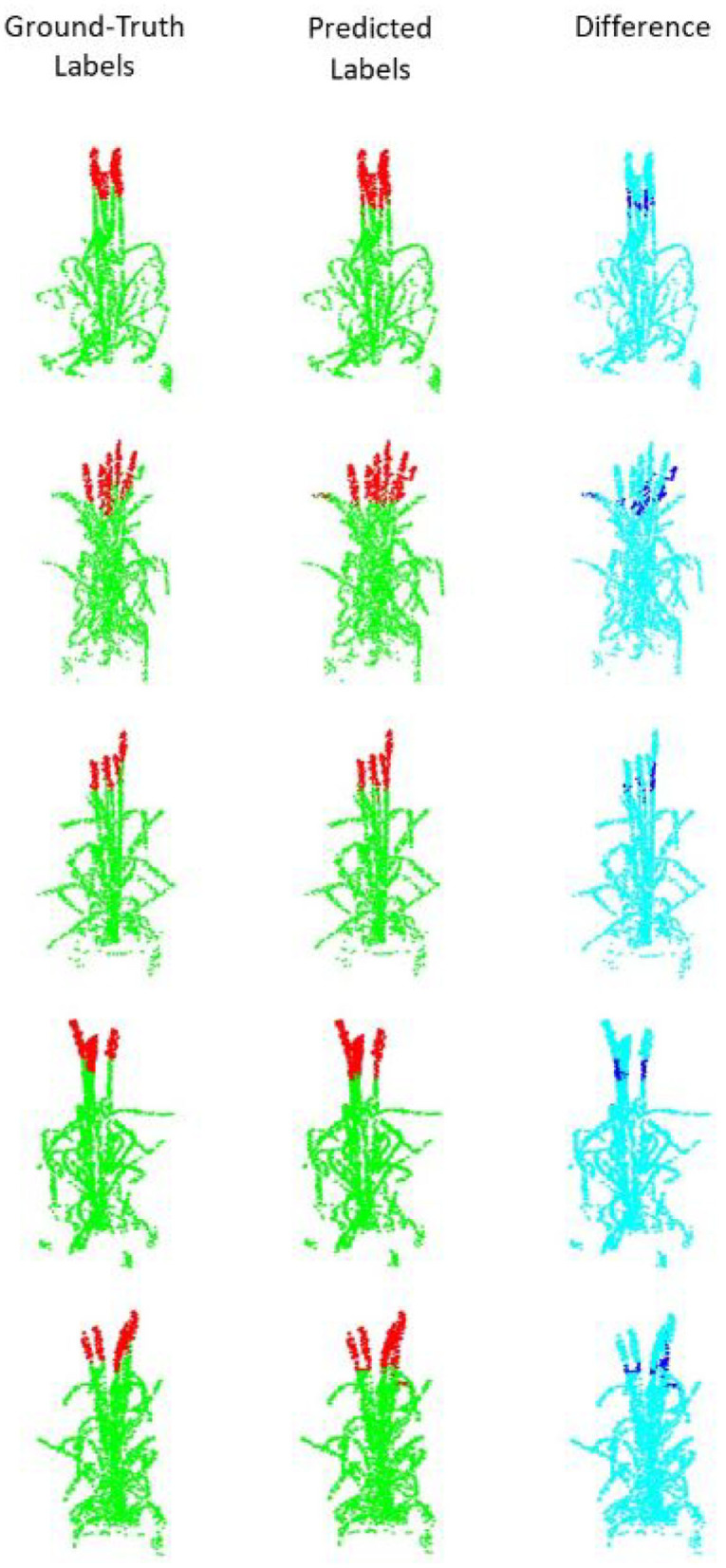
Segmentation results for point clouds containing 2,048 points. **(Left)** The ground truth samples (ears are shown in red and non-ears in green) annotated in MeshLab; **(middle)** The predicted/segmented labels; **(Right)** The difference between the ground truth and the segmented results is shown in dark blue.

The results are summarized in Table 1. The light Pattern-Net version works quite well but the most promising results are obtained by the Pattern-Net. Accuracy of above 91% indicates that when we increase the number of 3D points from 512 to 8,192, both the mean accuracy and the mean IoU results of the network are improved, as expected. Samples of results (Figure 4) show that the difference between the predicted labels and the reference mainly occurred in the border between the ear and the non-ear regions. This aspect of Pattern-Net is more favorable when we measure the dimension of attributes of interest. As seen in the table, the mean IoU of dimensions of segmented organs is above 80%. Deep learning-based networks can be improved by increasing the number of input samples. So, if one needs higher precision in the test experiments, then the network must be trained with additional relevant samples. We also carried out experiments for inputs with more than 8,192 points. To this end, we had to decrease the size of MLPs to half of the original because of a limitation in RAM available in our GPU. As shown in Table 1, the light Pattern-Net still works well with mean accuracy around 87% and achieving 88.13% mean accuracy for input point clouds of size 16,384 points. Typically, 16,384 points is considered to represent a dense model for plants with dimensions of <50 cm (height) × 50 cm (width) × 50 cm (length).

**Table 1:** Segmentation results of the proposed method on the wheat dataset.

**Network**	**#Input points**	**Mean accuracy**	**Mean IoU**
	**(xyz)**	**(*m*A)%**	**(*m*IoU) %**
Pattern-Net	512	91.17	80.19
	1,024	91.76	80.85
	2,048	92.07	81.26
	4,096	92.20	81.34
	8,192	92.27	81.74
Light Pattern-Net	10,240	87.32	76.47
	12,288	87.61	76.77
	14,336	87.97	77.12
	16,384	88.13	77.25

**Figure 5:**
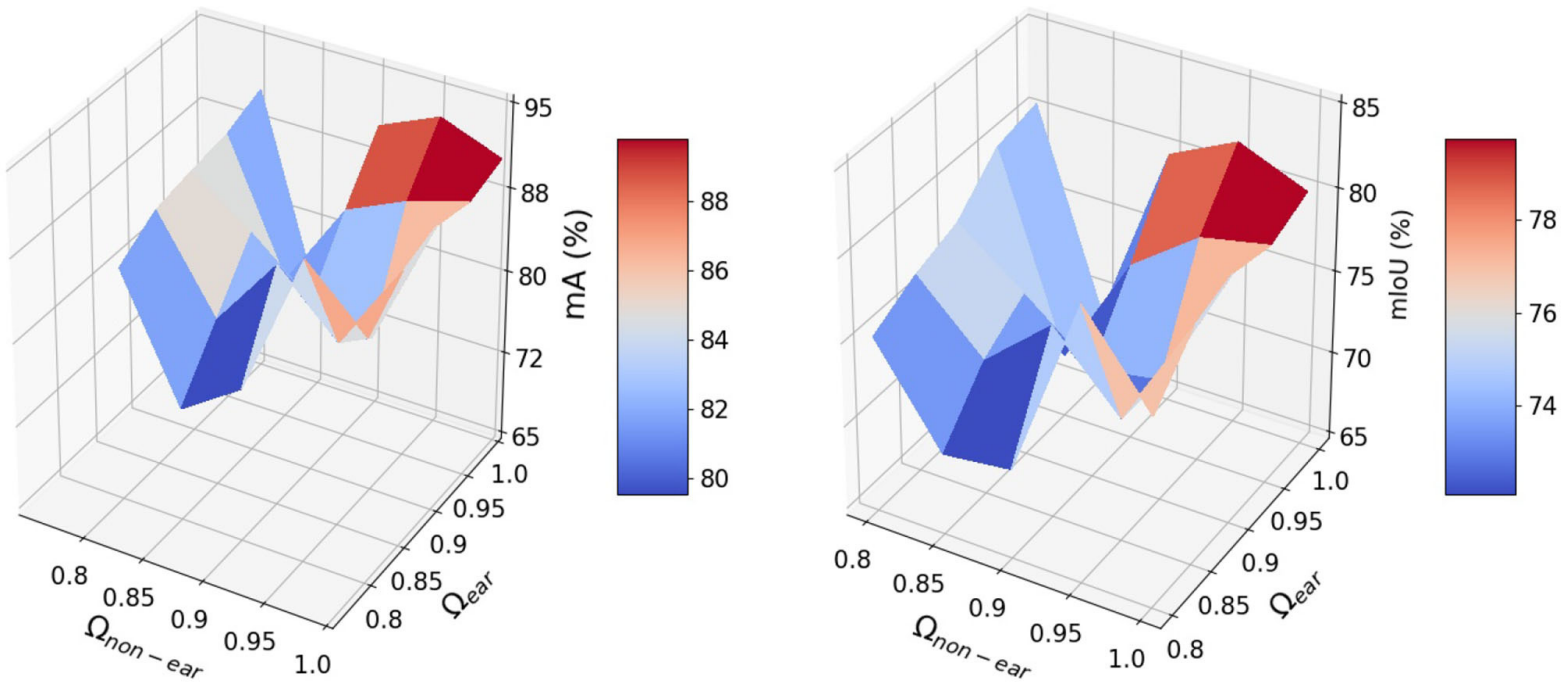
The influence of the dynamic coefficient Ω on the segmentation results.

**Figure 6:**
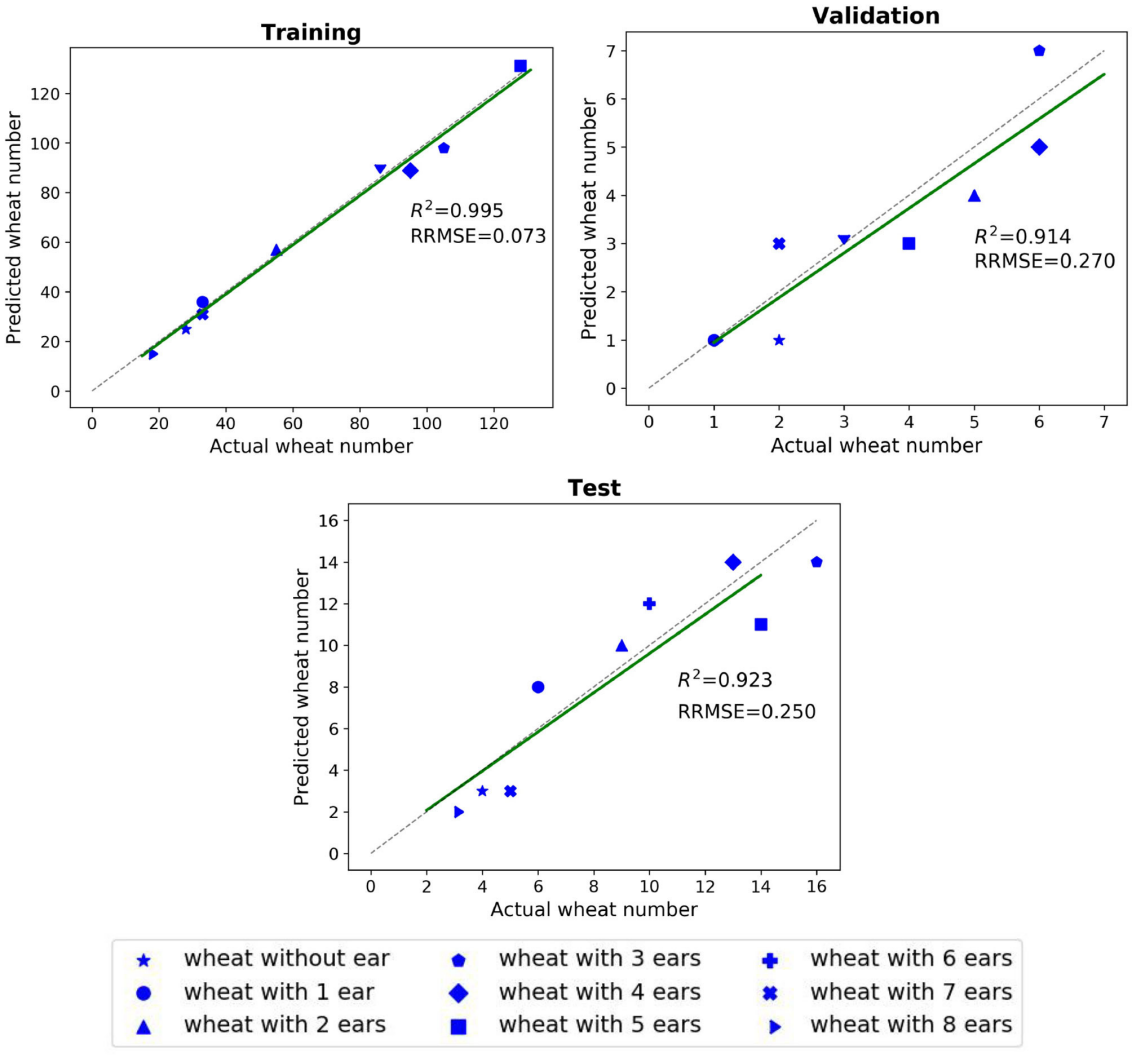
Comparison between the counted wheat samples with different ear numbers predicted by the Pattern-Net (vertical axis) and the ground-truth values (horizontal axis). We used MeshLab for collecting the ground-truth measurements in this experiment. The training, validation, and test experiments contain 580, 30, and 80 3D models with 1,024 points, respectively.

The impact of the coefficient vector, Ω, defined in Equation (9) is shown in Figure 5. The network works well when the weights are in the interval of [0.9,1] and achieves its best performance for *C*_*ear*_ = 1. and *C*_*non*−*ear*_ = 0.95. The dynamic coefficients balance between the loss of the majority non-ears points and that of the minority ear ones during training. Since vector Ω is a predetermined hyperparameter, we need to tune this parameter just once during training and the test step does not require the vector. The *R*^2^ and RRMSE results of the counted wheat samples with different ear numbers for training, validation, and test sets are reported in Figure 6. The ear number varies in the range of {0, 1, 2, …, 8}. The *R*^2^ results of the counted wheat between the automatic segmentation and the manually annotated ones in MeshLab are all higher than 0.91 and RRMSE all <0.3. The *R*^2^ result of the validation step is less than that of the test one due to the lower number of wheat samples, which is 30. The *R*^2^ of the counted wheat samples by Pattern-Net for the test dataset is more than 0.92, indicating the reliability of the proposed network in segmentation of the unseen test wheat samples.

### 4.5. The Manually Collected Post-data

**Figure 7:**
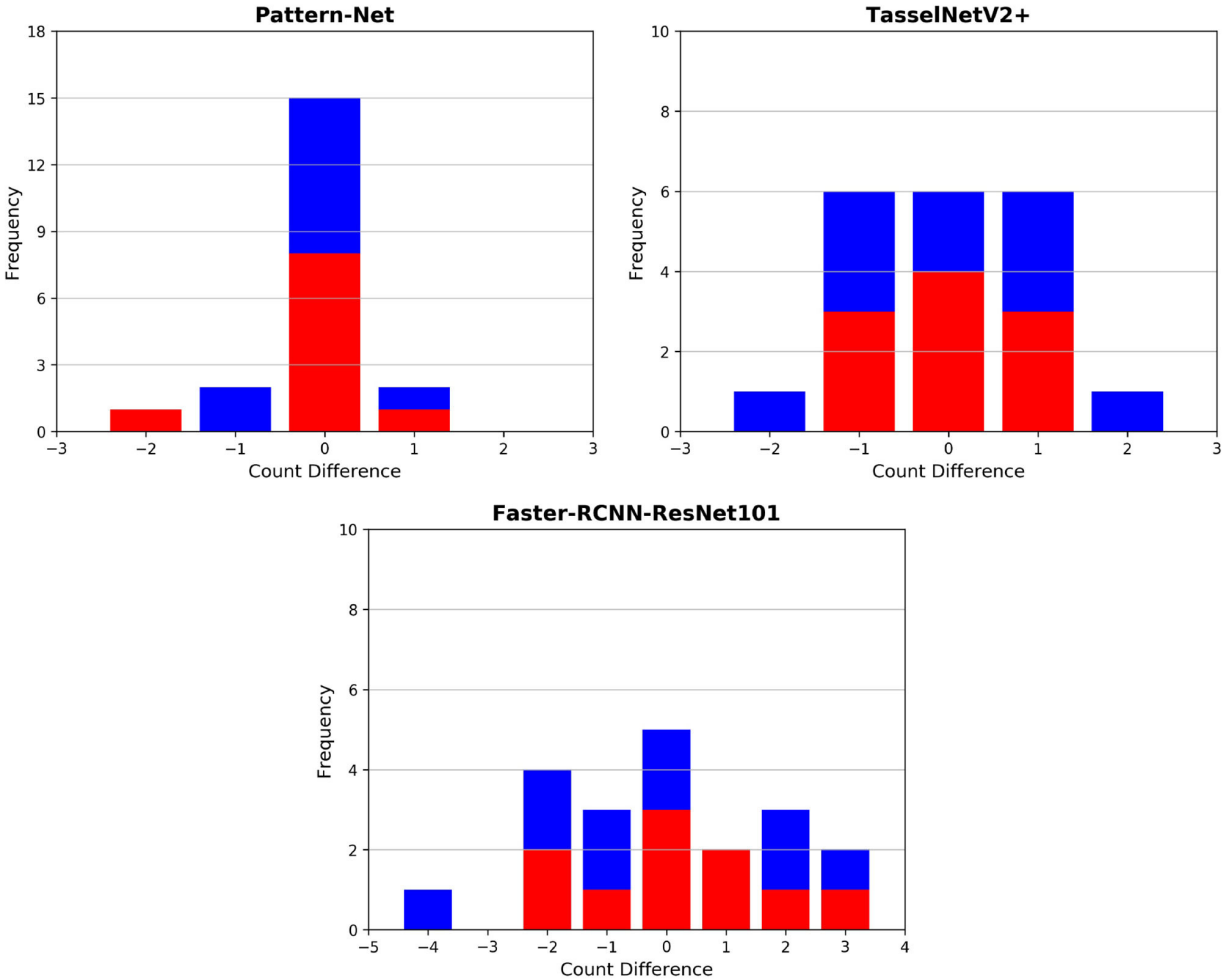
Histogram of count errors between the ears predicted by our 3D-based pipeline and 2D image-based approaches and the corresponding physical ground-truth measurements collected post-harvest; the image-based techniques include TasselNetV2+ (Lu and Cao, 2020) and Faster RCNN. The results of the individuals grown under well-watered and drought conditions are shown in red and in blue, respectively. 3D models with 2,048 points were used here. 2D images with 1,280 × 720 px were used for the image-based techniques.

**Figure 8:**
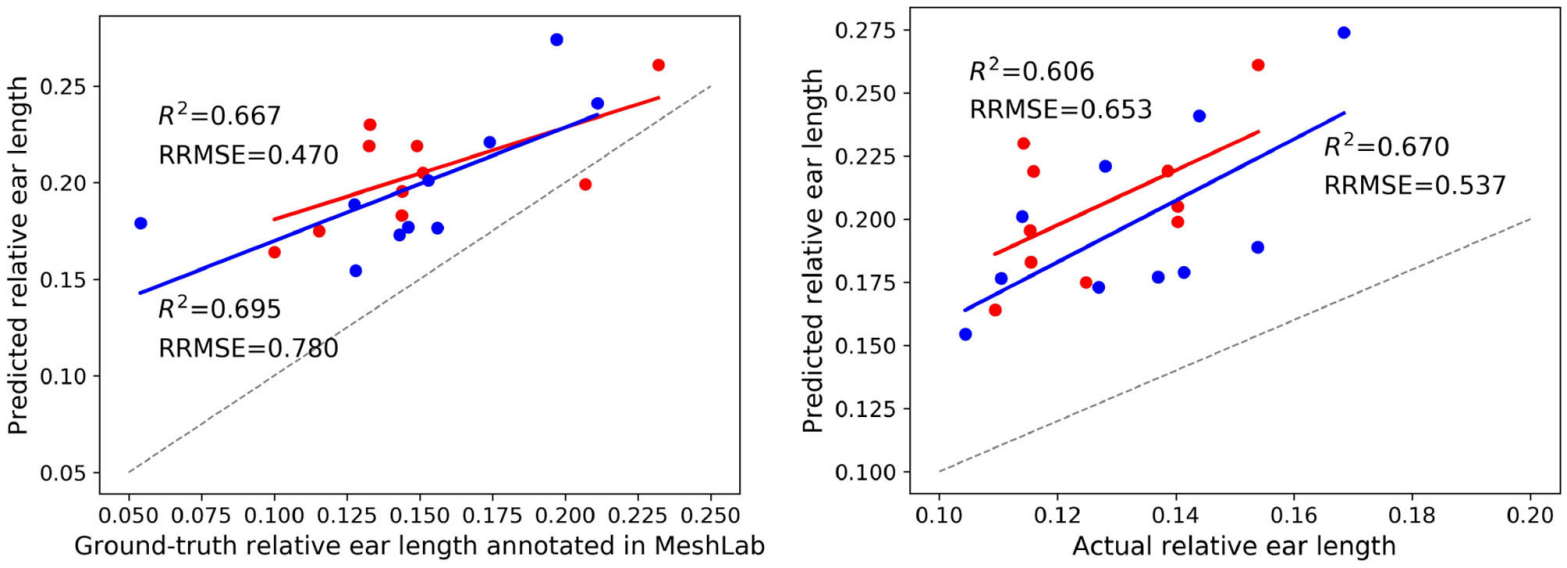
Results on the ear length of two treatments including 10 individuals grown under well water conditions (in red) and 10 individuals grown under drought conditions (in blue). **(Left)** Comparison between the average relative ear length identified by the Pattern-Net and the corresponding ground-truth values measured in MeshLab. **(Right)** Comparison between the relative ear length identified by the Pattern-Net and the corresponding physical ground-truth measurements collected post-harvest. 3D models with 2,048 points were used here.

Final post-harvest measurements of the two treatments for all 20 plants were collected manually. The difference between the predicted counted ears from Pattern-Net and the ground-truth data from physical post-harvest counting of ears was computed and the detailed distribution of errors is shown in Figure 7, where the ears of the most samples were counted correctly and the mean absolute difference of count errors is as low as 0.3. An important aspect of our method is that the length of ears was also predicted by the segmentation and their average results are shown in Figure 8. We collected the ground-truth values for ear length and plant height in MeshLab as well as by direct physical measurement of the plant material. For facilitating the comparison, the length of ears was normalized by the height of plants providing relative ear length. The *R*^2^ of average relative ear length between the segmentation and the actual ground-truth is 0.67 for the plants grown under drought conditions, which is on par with 0.695 of the ground-truth values annotated in MeshLab. The difference is as small as 0.025 and this figure for the plants grown under well water conditions is about 0.06. To determine the basis for differences between the MeshLab ground truth and the segmented results from Pattern-Net, we carefully compared the two and found that the classification of the border region between ear and non-ear regions could influence the predicted length of ears (Figure 4). Accurate classification of the border region remains a challenging task that needs further investigation.

## 5. Discussion

Geometrically accurate models of individuals that can be computationally interrogated would be of great value in quantifying and understanding phenotypic variation, both in fundamental biological studies as well in commercial production scenarios. Typical plants have a complex and variable body shape as well as a plastic developmental programme that can continue to alter their morphology across their entire life cycle. Their complex and variable shape present numerous challenges to building and analysing models at a speed and cost appropriate to their use, while progressive developmental change may necessitate repeated modeling of the same individual. The potential benefits of rapid cost-effective 3D modeling extend well beyond basic morphology, as many physiological processes also vary across the plant body, both spatially and temporally, so that emerging non-contact physiological assessment methods (Dieleman et al., 2019) often require complex correction for shape.

A number of different technologies have been developed, including LASER, Time of Flight, and LIDAR to capture information from living plants for modeling (Paulus, 2019). Medical imaging approaches, such as μCT scanning, have also been applied to plants, particularly for ears of wheat (Hughes et al., 2019) and analogous structures from other crops such as sorghum inflorescences (Li et al., 2020) but the trade-offs involved in image acquisition generally mean that the approach is applicable to either low numbers of complete plants or somewhat larger numbers of parts of plants. The capital investment in the scanning equipment is also substantial, putting this out of reach of most researchers. The image acquisition method we used is highly convenient in that it utilizes consumer-grade cameras and can be easily transferred to other labs and situations. The SfM method is widely used and the models produced are composed of 3D point clouds. These are a common format and there is much freely available software, such as MeshLab, for converting them into virtual objects with solid surfaces that then can be imported into CAD packages (for engineering, generally) or other analysis pipelines where features can be extracted, identified, and/or estimated. This approach works quite well for geometrically simple objects that generate clean simple models with relatively few outliers in the point cloud. However, plants are complex topologically and extensive occlusion tends to yield sub-optimal models that do not lend themselves to being converted to accurate surface-based models—on one hand, the outlying points tend to create spurious surfaces while on the other hand, occlusion and other imaging issues can lead to artifacts such as “holes” where there should be “tissue.” To solve these issues, various modifications to surface-based approaches have been developed with some success: we previously used a projection method to assess leaf angle during the imposition of drought stress in grapevines (Briglia et al., 2020). Pound et al. (2016) used an elegant patch and boundary-refinement method to reconstruct accurate models of wheat and rice leaves that they could extend to whole canopies.

However, Pattern-Net bypasses many of these issues by undertaking much of the analysis directly in the point cloud domain. Our results indicate that Pattern-Net can detect, classify, and measure features directly in the 3D point clouds with sufficient accuracy to compare with manual phenotyping. Also, and notwithstanding the current limitations on GPU resources, Pattern-Net can already be scaled to accommodate the analyses of many 100's of individual models. With access to more powerful facilities, we envisage that Pattern-Net would be capable of supporting longitudinal phenotyping of large genetically defined populations, such as MAGIC and diversity mapping populations (Camargo et al., 2016).

We and others have previously reported methods to produce models based on 3D point clouds and to identify biologically relevant features, including from wheat (Liu et al., 2018) and from diverse other species (Lou et al., 2014; Briglia et al., 2020). Different published ear detection methods compared with manual counting indicate Pattern-Net has a high level of correct feature identification (*R*^2^ > 0.9). Fernandez-Gallego et al. (2018) achieved correlations of up to *R*^2^ = 0.75 between their computer vision method using 2D images of field grown wheat and manual counting. Sadeghi-Tehran et al. (2019) used superpixels and CNN pretrained by a VGG16 model^5^ to achieve *R*^2^ of 0.94 on 126 test images. TasselNetV2+ (Lu and Cao, 2020) achieved *R*^2^ = 0.91 on the WEDD^6^ dataset (Madec et al., 2019). We tested TasselNetV2+ on our multi-view wheat samples. We used the pre-trained model released by the authors^7^ and the images were resized to 1,280 × 720 px. Since each sample consists of 74 multi-view images which are highly occluded, we ran TasselNetV2+ over all 74 images for each individual plant and took the maximum values as the predicted number of ears. The performance of TasselNetV2+ is shown in Figure 7. We also developed an image-based CNN using Faster RCNN ResNet101^8^. Faster RCNN was trained on the WEDD dataset. In both cases, the image-based techniques show lower accuracy compared to our 3D-based pipeline (Figure 7). The presence of occlusion in 2D images is inevitable, and the 3D-based pipeline can better deal with this problem. 3D models provide realistic depth that allows one to explore more accurately and enrich our understanding of the plant structures. The high cost of computing memory, however, is still a big challenge for processing in 3D space. Pattern-Net and its light version need 1.1*M* and 514*K* parameters, respectively. Our network gets to 92.3% test accuracy in 300 epochs of training, where the running-time for input 8,192 points is 253 seconds per epoch. The training time for the light Pattern-Net is 406 seconds per epoch for the input of 16,384 points.

It should be noted here that we used only a single variety of wheat, Paragon, whereas some of the 2D performance is given over many varieties and under less constrained imaging conditions (outdoors). Therefore, it is likely that Pattern-Net would require additional training before applying to other wheat cultivars or related cereals. Also, the definition of the boundary zone between ear and non-ear could be improved. This issue has arisen previously in the 2D analysis of rice panicles (the equivalent grain bearing structure to ears in wheat) and been solved by dual imaging with higher and lower resolution cameras followed by co-registration and a bespoke analysis pipeline (Huang et al., 2013). While many computer vision methods, in both 2D and 3D domains, can provide accurate feature recognition and counting, measurement of those features remains a challenge for plant phenotyping. We previously used an indirect RCNN to detect leaves in the 2D images projected from 3D point cloud models of grapevines subjected to drought and successfully quantified leaf angle to estimate a plant's response to stress (Briglia et al., 2020). Pattern-Net is capable of not only recognizing and counting ears accurately but also estimating their length, all within the 3D domain. Notwithstanding the issues associated with accurate recognition of the ear-non ear boundary in the point cloud, the output from Pattern-Net was well correlated (*R*^2^ > 0.6) with manual measurements for both well-watered and droughted plants. An innovation that may have helped modeling was additional viewpoints provided by the cameras. An interesting emerging approach is active imaging (Gibbs et al., 2018) where the camera(s), on a robotic arm, is relocated as required to overcome occlusion and to optimize the 3D model in a re-iterative manner. Such a system could be integrated into the conveyor system, in a similar manner to the dual-camera system used in this study. However, there are likely to be additional costs either in terms of image acquisition time, or computing power to ensure rapid real-time modeling and analysis.

To justify the additional costs, the 3D domain must add additional value and Pattern-Net begins to achieve this objective by providing quantification of a key morphological feature, ear length. This varies between cultivars and Siddique and Whan (1993) proposed that the ear to stem ratio might be a better indicator of yield potential than harvest index (HI) because the ratio is largely unperturbed by post-anthesis drought. They conceded that ear to stem ratio could only be used in early generations due to its labor-intensive data acquisition. Image-based approaches have the potential to reduce that labor burden, and Pattern-Net provides this metric as one of its outputs. As expected, the value of the ear: total plant height, manually measured or computationally inferred, increases slightly in the drought treatment and therefore Pattern-Net may be able to contribute to emerging Speed-Breeding (Watson et al., 2019).

## 6. Conclusion and Future Work

In this study, we have developed a CNN method for direct segmentation of 3D point clouds that is less susceptible to outliers. It is also invariant to changes in translation, rotation and scale. The key idea is to decompose the wheat point clouds into multiple subsets with similar structural information and then to force the network to learn and identify stable patterns. The network could successfully cope with the large-scale input point clouds ranging from 10,240 to 16,384 points and the results indicate that it is less prone to overfitting. This methodology provides a promising direction for robust analysis and understanding of plant point clouds although accurate estimation of ear length needs further improvement. While we have applied Pattern-Net to the relatively constrained datasets obtained from pot-grown wheat, this or similar approaches could be applied to field crops and canopies. The rapid and accurate assessment of the reproductive parts of many crops can be facilitated by image-based methods. For example, a dual-camera system has been developed for measuring harvested rice panicles (Huang et al., 2013). We expect that the principles developed within Pattern-Net can be applied to many other cereal crops, but in the context of intact plants.

## Data Availability Statement

The raw data supporting the conclusions of this article will be made available by the authors, without undue reservation.

## Author Contributions

MG, BT, YL, and JD designed the experiment. KW and FC undertook data acquisition, experimental design, and plant treatments. MG developed the deep learning and data analysis, testing and evaluation tasks, and drafted the manuscript. All authors have read and revised the manuscript.

## Conflict of Interest

The authors declare that the research was conducted in the absence of any commercial or financial relationships that could be construed as a potential conflict of interest.

## Footnotes

^1^http://www.fao.org/worldfoodsituation/csdb/en/

^2^3D CAD models are online available at http://modelnet.cs.princeton.edu

^3^https://www.plant-phenomics.ac.uk/

^4^https://www.gphoto.org/

^5^https://keras.io/api/applications/vgg/

^6^https://github.com/simonMadec/Wheat-Ears-Detection-Dataset

^7^https://github.com/poppinace/tasselnetv2plus

^8^https://docs.openvinotoolkit.org/2020.2/_models_intel_faster_rcnn_resnet101_coco_sparse_60_0001_description_faster_rcnn_resnet101_coco_sparse_60_0001.html
